# Foreign Body in the Throat

**Published:** 1899-02

**Authors:** Louis Brown


### Foreign Body in the Throat.

I had a very queer experience last Friday. A young man came to my office scarcely able to speak without a great deal of pain. He had been unable to eat since morning. There was apparently nothing the matter. He complained of soreness in his throat. He remarked that he had been perfectly well up until about 10 o'clock that morning, and had had no pain in his throat, but now was suffering desperately. In the morning he had gotten a little lunch, and amongst what he ate was a piece of sausage. From that on he felt something sticking. He thought he would eat a piece of bread, and if there was anything in his throat he would push it away, but nothing succeeded. I looked into his throat very carefully. The left tonsil was somewhat enlarged and a little redder than the one on the opposite side. I then illuminated his throat with an electric light, and I saw a little black speck, which would not move easily. I got hold of it with a fine pair of forceps and removed it. To my astonishment, I drew out a great big hog bristle seven-eighths of an inch long. How such a great bristle got into the sausage and avoided being cut by the sausage machine, is a mystery to me. Then, after getting through the sausage machine, how did it ever get into the tonsil? Under the microscope I found one end of the bristle cut exactly like a hypodermic needle. That was the end that entered the tonsil. What the patient suffered that day he described as something terrible, but after he lost his bristle he was all right.—*Dr. Louis Brown.*

